# Phenotypic and Transcriptomic Responses of *Campylobacter jejuni* Suspended in an Artificial Freshwater Medium

**DOI:** 10.3389/fmicb.2017.01781

**Published:** 2017-09-20

**Authors:** Hana Trigui, Kristen Lee, Alexandre Thibodeau, Simon Lévesque, Nilmini Mendis, Philippe Fravalo, Ann Letellier, Sébastien P. Faucher

**Affiliations:** ^1^Department of Natural Resource Sciences, Faculty of Agricultural and Environmental Sciences, McGill University, Sainte-Anne-de-Bellevue QC, Canada; ^2^Research Chair in Meat Safety, Department of Pathology and Microbiology, University of Montreal, Saint-Hyacinthe QC, Canada; ^3^Laboratoire de Santé Publique du Québec (LSPQ)/Institut National de Santé Publique du Québec, Sainte-Anne-de-Bellevue QC, Canada

**Keywords:** *C. jejuni*, microarrays, survival, oxidative stress, sodium choleate, starvation, cell wall

## Abstract

*Campylobacter jejuni* is the leading cause of campylobacteriosis in the developed world. Although most cases are caused by consumption of contaminated meat, a significant proportion is linked to ingestion of contaminated water. The differences between *C. jejuni* strains originating from food products and those isolated from water are poorly understood. Working under the hypothesis that water-borne *C. jejuni* strains are better equipped at surviving the nutrient-poor aquatic environment than food-borne strains, the present study aims to characterize these differences using outbreak strains 81116 and 81-176. Strain 81116 caused a campylobacteriosis outbreak linked to consumption of water, while strain 81-176 was linked to consumption of raw milk. CFU counts and viability assays showed that 81116 survives better than 81-176 at 4°C in a defined freshwater medium (Fraquil). Moreover, 81116 was significantly more resistant to oxidative stress and bile salt than strain 81-176 in Fraquil. To better understand the genetic response of 81116 to water, a transcriptomic profiling study was undertaken using microarrays. Compared to rich broth, strain 81116 represses genes involved in amino acid uptake and metabolism, as well as genes involved in costly biosynthetic processes such as replication, translation, flagellum synthesis and virulence in response to Fraquil. In accordance with the observed increase in stress resistance in Fraquil, 81116 induces genes involved in resistance to oxidative stress and bile salt. Interestingly, genes responsible for cell wall synthesis were also induced upon Fraquil exposure. Finally, twelve unique genes were expressed in Fraquil; however, analysis of their distribution in animal and water isolates showed that they are not uniquely and ubiquitously present in water isolates, and thus, unlikely to play a major role in adaptation to water. Our results show that some *C. jejuni* strains are more resilient than others, thereby challenging current water management practices. The response of 81116 to Fraquil serves as a starting point to understand the adaptation of *C. jejuni* to water and its subsequent transmission.

## Introduction

*Campylobacter jejuni* is the leading cause of bacterial food-borne diarrheal disease in the developed world (Dasti et al., 2010). Acute *C. jejuni* infection causes watery to bloody diarrhea, with fever, nausea, and vomiting, and can be fatal to vulnerable individuals (Butzler and Skirrow, 1979; Walker et al., 1986). Although the infection is often self-limiting, it has been reported to lead to the development of secondary autoimmune disorders such as the Guillain-Barré or Miller-Fisher syndromes (Wassenaar and Blaser, 1999; Young et al., 2007). *C. jejuni* is a prevalent human pathogen but is usually viewed as commensal in livestock, particularly in poultry (Inglis and Kalischuk, 2004; Boes et al., 2005; Gormley et al., 2008). The majority of human infections occur directly through consumption of raw or undercooked contaminated animal products, such as meat and milk, or indirectly through cross-contamination events in the consumer kitchen. Nevertheless, animal products are not the sole route of transmission of *C. jejuni* to humans. Analysis of waterborne outbreaks and sporadic cases show that water is an important environmental reservoir for *C. jejuni* (Bolton et al., 1982; Thomas et al., 1999; Huang et al., 2015). Contamination of surface water and well water may occur due to direct deposition of animal feces, sewage discharge and farmland run-offs (Vogt et al., 1982; Lind et al., 1996; Clark et al., 2003; O’Reilly et al., 2007; Bronowski et al., 2014; Huang et al., 2015).

*Campylobacter jejuni* is a microaerophilic bacterium that grows best at temperatures ranging from 37 to 42°C, and requires a rich growth medium (Skirrow, 1991). In the host digestive tract, *C. jejuni* encounters various challenges, such as acidity, antimicrobial bile salts, resident microorganisms, fluctuations in osmolarity, and effectors of the immune system (Fordtran and Locklear, 1966; Stintzi et al., 2005). Once expelled from one host, *C. jejuni* is exposed to and must survive a different set of stress conditions before colonizing another host (Bronowski et al., 2014). The conditions encountered within the different transmission routes are variable; transmission to humans through contaminated meat subjects the bacterium to stresses that are different from those found when its transmission occurs through water (Bronowski et al., 2014). Potential stresses encountered in water include nutrient scarcity, extreme temperatures, disinfectant and osmotic stresses (Thomas et al., 1999; Murphy et al., 2006; Jackson et al., 2009). *C. jejuni* must therefore overcome these challenges to survive and use water as a transmission route. Presumably, strains that survive the best in water are the most likely to be succesfully transmitted between hosts through this medium. Supporting this hypothesis, some *Campylobacter* multilocus sequence type (ST) complexes (ST-2381, ST-45, and ST-1225) were found to be more commonly associated to water (Sopwith et al., 2008; Carter et al., 2009). The high incidence of ST-45 in river water isolates and in human infections could indicate that it is well adapted to environmental transmission routes (Murphy et al., 2005; Lévesque et al., 2013). Indeed, *C. jejuni* strains assigned to ST-45 survive heat, aerobic and oxidative stresses better than other sequence types (Habib et al., 2010).

The multiple transmission routes of *C. jejuni* suggest that some strains may possesses effective mechanisms that allow it to sense and cope with a variety of stresses present in a given niche (Bronowski et al., 2014). Contributing to the survival success of *C. jejuni* is its ability to persist in natural environments by adapting lifestyles other than the planktonic form. Consequently, *C. jejuni* can be found as a free-living member of complex multispecies biofilm (Nguyen et al., 2011), internalized within some waterborne protozoa (Axelsson-Olsson et al., 2005; Snelling et al., 2005; Baffone et al., 2006), excreted within multilamellar bodies (MLBs) by ciliates (Trigui et al., 2016) and viable but non-culturable (VBNC) cells (Rollins and Colwell, 1986; Murphy et al., 2006). Given that water is a vehicle for the spread of *C. jejuni*, many studies have focused on the survival of *Campylobacter* in different types of water, such as tap water (Buswell et al., 1998; Cools et al., 2003), bottled mineral water (Tatchou-Nyamsi-König et al., 2007), artificial seawater (ASW) (Baffone et al., 2006; Trigui et al., 2015b) and a defined freshwater medium (Fraquil) (Trigui et al., 2015b). Notably, several *Campylobacter* strains were found to enter the VBNC state after exposure to the aforementioned water sources (Cools et al., 2003; Baffone et al., 2006; Trigui et al., 2015b). VBNC *C. jejuni* cells were able to maintain their ability to adhere to intestinal cells after 3 weeks in freshwater at 4°C (Patrone et al., 2013). In addition, *Campylobacter*-naïve chicks that consumed water contaminated by VBNC *C. jejuni* were successfully colonized by the bacterium (Pearson et al., 1993). Therefore, VBNC *C. jejuni* are considered a threat to public health (Murphy et al., 2006).

Some of the *C. jejuni* stress response mechanisms and their regulators that have been studied to-date are distinct from those in other enteric Gram-negative pathogens, while others remain poorly understood. For example, full genome analyses of *C. jejuni* strains suggest that this pathogen has a relatively small genome (Parkhill et al., 2000) and lacks many classical stress tolerance regulators, such as the stationary phase sigma factor RpoS, the oxidative stress response regulators SoxRS and OxyR, and the osmotic shock regulator BetAB (Murphy et al., 2006). In contrast, genes related to iron metabolism and oxidative stress defense, which are controlled by the ferric uptake regulator (Fur) and the peroxide responsive regulator (PerR), respectively, are key factors for *C. jejuni*’s survival *in vivo* (Palyada et al., 2009; Flint et al., 2014; Butcher et al., 2015).

Recently, we investigated the survival of *C. jejuni* chicken cecal isolates in Fraquil, artificial sea water and Fraquil supplemented with salt (Trigui et al., 2015b). Fraquil is an artificial freshwater medium used to study the behavior of bacteria in water (Mendis et al., 2015). The strains tested varied significantly in their ability to survive in the three aforementioned water systems, presumably due to genetic differences between the isolates. In the present study, the survival of two additional model strains was evaluated in Fraquil. Strain 81116 remained culturable and viable longer than strain 81-176 in Fraquil. Moreover, strain 81116 was more resistant to oxidative stress and exposure to bile salts after incubation in water relative to 81-176. Given that 81116 was better adapted to surviving this aquatic environment, we performed a microarray analysis to uncover its transcriptomic response when exposed to water.

## Materials and Methods

### Bacterial Strains and Media

The *C. jejuni* strains used in this study are listed in **Table 1**. *C. jejuni* was stored at -80°C in Brucella broth containing 10% glycerol. *C. jejuni* was routinely grown on TSA-blood plates (1.5% pancreatic digest of casein, papaic digest of soybean, 0.5% sodium chloride, 1.5% agar and 5% defibrinated sheep blood) for 2 days at 42°C under a microaerophilic atmosphere generated with the CampyGen system (Oxoid). For liquid culture, *C. jejuni* was grown in Brucella broth (BD Biosciences) containing 10 g/L pancreatic digest of casein, 10 g/L peptic digest of animal tissue, 1 g/L dextrose, 2 g/L yeast extract, 5 g/L sodium chloride, 0.1 g/L sodium bisulfite.

**Table 1:** Strains used in this study.

Name	Origin	Condition of isolation	Reference
81116	Human	Clinical isolate (water-borne outbreak)	Korlath et al., 1985
81-176	Human	Clinical isolate (raw milk-borne outbreak)	Palmer et al., 1983
NCTC11168_H	Human	Clinical isolate	Ahmed et al., 2002
RM1221_C	Chicken	Store-bought chicken carcass	Miller et al., 2000
L2003a_C	Chicken	Caecal content at time of slaughter	Thibodeau et al., 2015
T2003a_C	Chicken	Caecal content at time of slaughter	Thibodeau et al., 2015
D2008aC	Chicken	Caecal content at time of slaughter	Thibodeau et al., 2015
F2008d_C	Chicken	Caecal content at time of slaughter	Thibodeau et al., 2015
F2008a_C	Chicken	Caecal content at time of slaughter	Thibodeau et al., 2015
G2008b_C	Chicken	Caecal content at time of slaughter	Thibodeau et al., 2015
A2008a_C	Chicken	Caecal content at time of slaughter	Thibodeau et al., 2015
006A0089_B	Bovine	Fresh feces sample picked at the farm	Lévesque et al., 2013
007A0289_W	Water	Environmental surface water	Lévesque et al., 2013
007A0333_W	Water	Environmental surface water	Lévesque et al., 2013
007A0418_W	Water	Environmental surface water	Lévesque et al., 2013
007A0613_W	Water	Environmental surface water	Lévesque et al., 2013
007A1045_W	Water	Environmental surface water	Lévesque et al., 2013
007A1078_W	Water	Environmental surface water	Lévesque et al., 2013
007A1431_W	Water	Environmental surface water	Lévesque et al., 2013
012A0093_SG	Snow Goose	Fresh feces sample picked from the soil	Lévesque et al., 2013
012A0094_G	Gull	Fresh feces sample picked from the soil	Lévesque et al., 2013

### Survival in Fraquil

The survival of *C. jejuni* strains 81116 and 81-176 was evaluated in the artificial freshwater media Fraquil as described previously (Trigui et al., 2015b). The composition of Fraquil is 0.004% (wt/vol) CaCl_2_, 0.004% MgSO_4_, 0.001% NaHCO_3_, 0.0002% K_2_HPO_4_, 0.004% NaNO_3_, 10 nM FeCl_3_, 1 nM CuSO_4_, 0.22 nM (NH_4_)_6_Mo_7_O_24_, 2.5 nM CoCl_2_, 23 nM MnCl_2_ and 4 nM ZnSO_4_ (Morel et al., 1975). Bacteria grown on agar plates were suspended in Fraquil in a 5 ml plastic tube (Sarstedt), washed three times with Fraquil, and the optical density was adjusted to 0.1 at 600 nm (OD_600_). Centrifugation for the washing steps were performed at 5,000 *g* for 10 min at room temperature. The suspensions were then further diluted 1:5 in Fraquil, and then incubated at 4°C or 25°C. CFU counts were determined periodically on TSA-blood plates.

### LIVE/DEAD Staining

The BacLight^TM^ LIVE/DEAD^®^ bacterial viability kit (Life Technologies) was used to stain *C. jejuni* in Fraquil according to the manufacturer’s protocol. A Guava easyCyte flow cytometer (EMD Millipore) was used to analyze stained cells as described previously (Trigui et al., 2015a). Sterile Fraquil containing the LIVE/DEAD stain was used as a blank. Freshly cultured *C. jejuni* was used as the live control and *C. jejuni* incubated in boiling water for 10 min was used as the dead control for data analysis. Controls were performed for each strain. Both controls and samples were diluted to an OD_600_ of 0.01 before staining and analysis by flow cytometry.

### Stress Resistance Tests

The procedure to test the sensitivity of *C. jejuni* to sodium hypochlorite, hydrogen peroxide and sodium choleate were adapted from a previous study (Levi et al., 2011). Briefly, one milliliter aliquots of 81116 and 81-176 were retrieved from 4h-old Fraquil or Brucella broth suspensions incubated at 4°C and transferred to a 24-well plate (Sarstedt). Each strain was treated in triplicate for 1 h with 500 μM of H_2_O_2_ (Sigma–Aldrich) or 100 mg/ml of Na-choleate (Sigma–Aldrich). For the sodium hypochlorite test, Clorox bleach solution containing 10.3% of sodium hypochlorite was added to the wells at different final concentrations (0.0001, 0.00013, 0.0002, and 0.0003%). No treatment controls were also included. The samples were incubated at 4°C for 1 h prior to plating on TSA-blood agar plates for CFU enumeration. The differences in CFU counts between the controls and the treatments were calculated for each strain and stress condition.

### Transcriptomic Analysis by Microarray

Strain 81116 cultured on TSA-Blood agar at 42°C for 2 days was suspended in 100 ml of Fraquil or Brucella broth at an OD_600_ of 1 in triplicate, and washed three times with either Fraquil or Brucella broth, respectively. The suspensions were then incubated at 4°C for 4 h. Samples for RNA extraction, Live/Dead staining and CFU count were collected from each replicate. For RNA extraction, the cells were pelleted by centrifugation, suspended in 40 μl of Tris-EDTA, and lysed by the addition of 1 ml of TRIzol reagent. RNA extraction was performed with TRIzol reagent according to the manufacturer’s protocol. The RNA was subsequently treated with Turbo DNase (Ambion) and purified by acid-phenol extraction. The purity and concentration of RNA were determined by UV spectrophotometry. The integrity of extracted RNA was confirmed on a formaldehyde-agarose gel. 15 μg of RNA was labeled with amino-allyl dUTP (Sigma) during reverse transcription (Superscript II; Invitrogen) using random hexamers (Invitrogen) as previously described (Hovel-Miner et al., 2009; Faucher and Shuman, 2013). Genomic DNA was used as a reference channel and labeled by random priming using Klenow fragments, amino-allyl dUTP, and random primers as described previously (Faucher and Shuman, 2013). DNA was subsequently coupled to the succinimidyl ester fluorescent dye (Invitrogen) Alexa Fluor 647 (for cDNA) or Alexa Fluor 546 (for gDNA) according to the manufacturer’s protocols.

The microarray slides designed and produced by Mycroarray for *C. jejuni* strain 81116 was used (GEO accession numbers GPL23071). Pre-hybridization, hybridization and washing were carried out as described previously (Trigui et al., 2015a). Data acquisition was performed with an InnoScan 710 microarray scanner and data analysis was performed as previously described (Trigui et al., 2015a). Background signal was subtracted and the ratio between Fraquil and broth (F/B) was calculated for each probe. The ratio was considered differentially expressed when the log2 ratio was higher than 1 or lower than -1, and the student’s *t*-test *P*-value was lower than 0.05. The complete dataset was deposited in GEO (GSE94930).

### Reverse Transcription-Quantitative PCR (RT-qPCR)

RNA was extracted and purified from 81116 exposed to Fraquil or Brucella broth for 4 h at 4°C. Three biological replicates were tested. One μg of RNA was used for reverse transcription reactions along with a negative control without reverse transcriptase. qPCR was performed on an iQ^TM^5 Multicolor Real-Time PCR Detection System (Bio-Rad) using iTaq universal SYBR green supermix (Bio-Rad) according to manufacturer’s protocol. Gene-specific primer sets were designed with the IDT primer design software (Bachman and Swanson, 2001) (**Table 2**) and their amplification efficiency was determined experimentally to be >85%. The 16S rRNA gene was used as a reference to normalize the data. Fold change was calculated as described previously (Livak and Schmittgen, 2001) and presented as a log2 ratio.

**Table 2:** Primers used in this study.

Gene	Primer name	Primer sequence
C8J_0133	C8J0133-F	TATTGCTGGGCATAGGAAAGG
	C8J0133-R	TCTAGCAGCTTCTCTTGGAGTA
C8J_0398	C8J0398-F	GCAACATCTACCGTGATGCTAA
	C8J0398-R	ACATATCTACAATCCACCAAATCCA
C8J_0648	C8J0648-F	GTATCAGCAGACATAAGACAAGG
	C8J0648-R	TGCTTTCTTCTAGGTACTCTTTATC
C8J_1333	C8J1333-F	TGAGCTTGCACAAGATGATACC
	C8J1333-R	GCACCAGAATACAAACCCTTCT
C8J_1342	C8J1342-F	GTTGATTTAGTGGCAGTTGGTG
	C8J1342-R	CTCTTTCTACTGCTCCTTGAATACT
C8J_1423	C8J1423-F	AAATTTATGCGCGTGCTTT
	C8J1423-R	AACTATGCCACCAAGCAAA
C8J_1619	C8J1619-F	CCAAAGTGGATAGTATTGCAAGAATTAG
	C8J1619-R	GACGACTTAAAGAACTTGAAACTGG
frdA	qfrdA-F	GTGTGCCTTGGACTAGAGTTAC
	qfrdA-R	CTGCGATATAGCAAGTTCTCCA
ccpA-2	qccpA-2-F	GTGGTATCATTTCTTGTAATACCTGTC
	qccpA-2-R	TGATGAGGATTTGCTGTCCAT
racR	qracR-F	ACGGATACAGCGTTTCAAGAG
	qracR-R	ACTCTTAAGCGACCGATGATAAC
flhB	qflhB-F	GGAAGGAGATCCTCAGGTTAAAG
	qflhB-R	GCATAATGCGTTGGGTTTGT
kpsM	qkpsM-F	TGTGGAACCTTTAAGAACTTTGC
	qkpsM-R	AAGCAAAGGACGAGGAGTTAG
cmeB	qcmeB-F	GCCATAGGGATCGTTGTAGATG
	qcmeB-R	CTATCCAAGCGATGCAAGAAGT
16S rRNA	16S-qF	AGAGATGCATTAGTGCCTTCGGGA
	16S-qR	ACTAAGGATAAGGGTTGCGCTCGT

### Distribution of the Unique Genes of 81116 in *C. jejuni* Isolates

The presence of genes unique to 81116 and expressed in Fraquil was evaluated by PCR. Genomic DNA was isolated from *C. jejuni* using the Wizard Genomic DNA Purification Kit. Primer sets were designed with IDT-PrimerQuest (**Table 2**). PCR was performed on 10 ng of gDNA using OneTaq polymerase (NEB). The PCR products were analyzed on a 0.7% agarose gel. Strains 81116 and 81-176 served as positive and negative controls, respectively.

## Results

### Comparative Survival of *C. jejuni* 81116 and 81-176 in Fraquil

**FIGURE 1:**
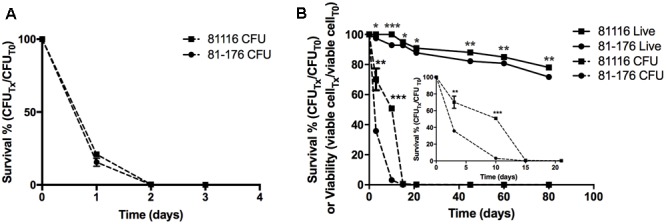
Survival of 81116 and 81-176 in Fraquil. Strains 81116 (square) and 81-176 (circle) were grown on TSA-blood, suspended in Fraquil and incubated at 25°C **(A)** and 4°C **(B)**. The number of cells was evaluated by CFU counts (dashed line). The viability of each strain (solid line) was evaluated using the LIVE/DEAD staining kit (Invitrogen) and flow cytometry. We used an unpaired Student’s *t*-test to assess statistical significance for 81116, vs. 81-176 (^∗^*P* ≤ 0.05, ^∗∗^*P* ≤ 0.005, ^∗∗∗^*P* ≤ 0.0005).

Here, we compared the survivorship in Fraquil of two widely used reference strains, 81116 and 81-176 which were originally isolated from two human outbreaks. Strain 81116 was the etiological agent of a water-borne outbreak (Palmer et al., 1983) and strain 81-176 caused an outbreak due to consumption of raw milk (Korlath et al., 1985). Given its origin from a water-source, we hypothesized that strain 81116 would better retain viability and culturability in water compared to strain 81-176. To this end, 81116 and 81-176 were suspended in Fraquil and incubated at 25°C and 4°C, the refrigeration temperature known to favor the survival of *C. jejuni* in water (Buswell et al., 1998; Thomas et al., 1999; Tatchou-Nyamsi-König et al., 2007; Trigui et al., 2015b). As expected, both strains showed a steep decline at 25°C (**Figure 1A**). At 4°C, 81116 survived better in Fraquil than 81-176 (**Figure 1B**). After 10 days in water, 50% of the 81116 population were culturable compared to only 3% of the 81-176 population. By day 21, the percent culturability was 0.2 and 0.003% for 81116 and 81-176, respectively, falling to 0% thereafter. To determine whether loss of culturability on agar plates was due to cell death, the viability of each population was assessed using the LIVE/DEAD kit and flow cytometry, as previously described (Trigui et al., 2015b). Freshly grown *C. jejuni* was used as a live control, while heat-killed *C. jejuni* served as a dead control. In contrast to the sharp decline in culturable cells, the viability of the *C. jejuni* strains decreased slowly over time. Nonetheless, 81116 showed a small but significantly higher viability compared to 81-176 (**Figure 1**). It is not clear whether incubation in water for 80 days produced authentic viable-but-non-culturable cells, since resuscitation was not attempted. Nevertheless, 81116 survived in Fraquil better than 81-176.

### 81116 Is More Resistant Than 81-176 to a Variety of Stresses in Fraquil

Adaptation to the low nutrient content of Fraquil could mediate cross-adaptation to other stresses. Indeed, starved *Escherichia coli* cells are more resistant to osmotic stress and oxidative stress (Jenkins et al., 1988, 1990). We investigated whether a similar adaptation occurred in *C. jejuni* after a short-term exposure to Fraquil, compared to short-term exposure to rich broth. Since chlorine and other oxidative disinfectants are routinely used to control the presence of *C. jejuni* in potable water and in slaughterhouse water chillers (Kameyama et al., 2012), the resistance of 81116 and 81-176 toward hydrogen peroxide and sodium hypochlorite after exposure to Fraquil was investigated. During the infection process, *C. jejuni* is exposed to bile salts in the small intestine (Begley et al., 2005). The *C. jejuni* capsule increases resistance to bile salts, but also contributes to avoiding complement-mediated killing, increasing bacterial colonization and bacterial persistence within the chicken host (Wong et al., 2015). As such, an increased resistance to bile salts after water exposure may indicate a strain’s host colonization potential. Therefore, the resistance of 81116 and 81-176 to sodium choleate, containing the main constituents of bile (Begley et al., 2005), was also tested. To determine the relative resistance of *C. jejuni* when faced with the aforementioned stresses, 81116 and 81-176 were suspended in Fraquil and rich broth, and incubated for 4 h at 4°C. Each strain were then added to the suspension and CFUs were determined by serial dilution and plating on TSA-blood plates before adding the stresses and after 1 h of exposure to the different stresses.

**FIGURE 2:**
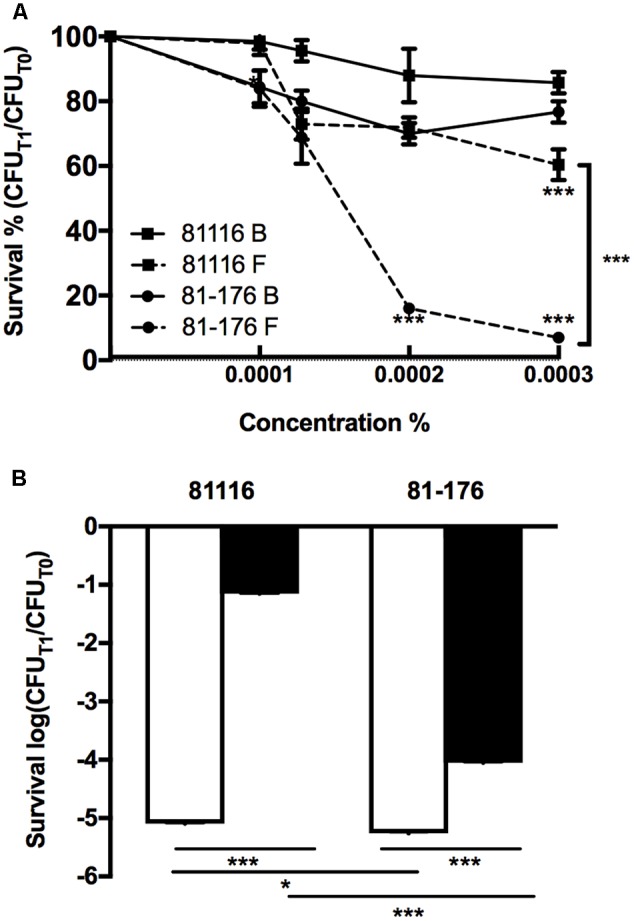
Resistance of 81116 and 81-176 strains to oxidative stresses. The strains were suspended in Brucella broth (B, white bar) and Fraquil (F, black bar) for 4 h at 4°C and then, exposed to **(A)** a dilution series of sodium hypochlorite and **(B)** 500 μM hydrogen peroxide (H_2_O_2_). CFU counts were determined before exposure to stress (CFU_T0_) and after 1 h of treatment (CFU_T1_). Error bars represent the standard deviation from three independent biological replicates. An unpaired Student’s *t*-test was used to assess statistical significance between broth and Fraquil in **(A)**, unless noted otherwise, and between each condition for **(B)** (^∗^*P* ≤ 0.05, ^∗∗^*P* ≤ 0.005, ^∗∗∗^*P* ≤ 0.0005).

Sodium hypochlorite had little effect on the survival of the strains suspended in broth. However, 81-176 suspended in Fraquil showed a marked decreased in survival with increasing sodium hypochlorite concentration (**Figure 2A**, circles), whereas, the survival of 81116 was only slightly affected by sodium hypochlorite exposure in water (**Figure 2A**, squares). Upon exposure to hydrogen peroxide in rich broth, the CFU counts of both strains decreased by 5 logs after 1 h (**Figure 2B**). In contrast, the strains suspended in Fraquil showed a greater resistance; the CFU counts of 81-176 decreased by 4 logs, while 81116 showed a mere 1 log decrease in CFUs. Overall, these results suggest that 81116 is more resistant to oxidative stress than 81-176 when suspended in Fraquil, but both strains exhibit a similar sensitivity to hydrogen peroxide in Brucella broth.

**FIGURE 3:**
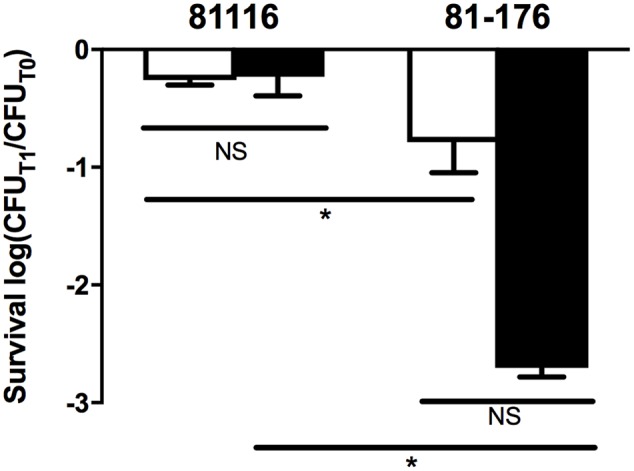
Resistance of 81116 and 81-176 to sodium choleate. The strains were suspended in Brucella broth (white bars) and Fraquil (black bars) for 4 h at 4°C and then exposed to 100 mg ml^-1^ of sodium choleate. CFU was determined before exposure to stress (CFU_T0_) and after 1 h of treatment (CFU_T1_). Error bars represent the standard deviation from three independent biological replicates. An unpaired Student’s *t*-test was used to assess statistical significance (NS, not significant, ^∗^*P* ≤ 0.05).

Strain 81-176 was also significantly more sensitive to sodium choleate relative to strain 81116. While the latter strain showed no difference in its survival when challenged with sodium choleate in broth compared to the same stress in water, 81-176 was almost 100-fold more sensitive to bile salts in water relative to the rich medium, however this difference was not statistically significant (**Figure 3**). As such, we conclude that 81116 is more resistant than 81-176 to bile salt.

### Transcriptomic Response of 81116 to Fraquil

**FIGURE 4:**
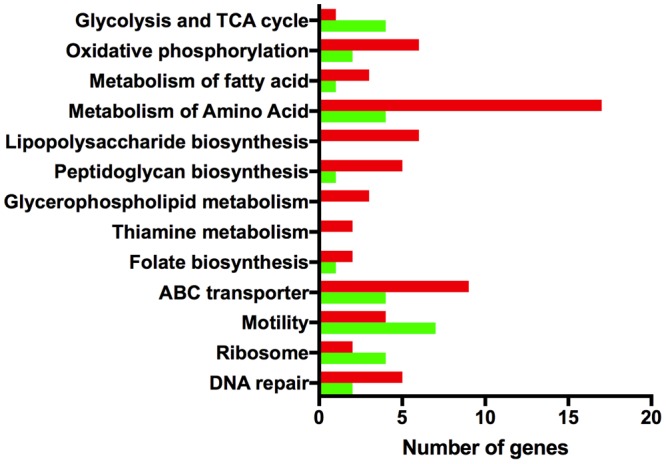
Gene ontology analysis of differentially expressed genes. The Kyoto Encyclopedia of Genes and Genomes (http://www.genome.jp/kegg/kegg2.html) BRITE Hierarchy was used to classify differentially expressed genes into categories. The *x*-axis shows the number of genes that are induced (red) and repressed (green) in each group.

Taken together, the phenotypic analysis indicates that strain 81116 is better equipped to induce a genetic response that promotes its survival in Fraquil. Therefore, the transcriptomic response of this strain in response to Fraquil was elucidated using microarray analysis. To this end, 81116 was exposed to either Fraquil or Brucella broth (rich medium) for 4 h at 4°C, in triplicate. RNA was extracted and the transcriptomic profiles were analyzed using DNA microarrays. To identify the genes differentially expressed in water, the transcriptome in Fraquil was compared to that in broth (F/B). In order to ensure reliable quantitative measurements of gene expression, most genes were represented by three different, non-overlapping probes. A few genes could only accommodate one or two probes, because of their small size or homology with other genes. Genes were considered differentially expressed when the following conditions were met; (1) all probes show a twofold change in the same direction and (2) at least 50% of the probes have a *P-*value less than 0.05. Differentially expressed genes were classified into Gene Ontology (GO) groups according to their cellular functions (**Figure 4**). **Table 3** contains the fold-change expression of selected genes and the complete dataset is presented in Supplementary Table S1. Exposure to Fraquil leads to the induction of 187 genes and the repression of 149 genes. *cmeB*, coding for an efflux pump, and *ccpA-2*, coding for a cytochrome peroxidase, are among the stress response genes that are induced in 81116 upon exposure to Fraquil. Genes involved in enterobactin uptake, such as *ceuDE* and *cfrB*, were also strongly induced in 81116. Notably, many genes involved in the metabolism of amino acids, including *aspA, sdaA, glnA*, and *ggt* are repressed in water (**Figure 4**).

**Table 3:** Select genes differentially expressed in water.

Gene	Name	Product	F/B (log2)
**Amino acid transporter**			
C8J_0858	*pebA*	Amino acid ABC transporter, periplasmic amino	–5.08
C8J_0859	*pebC*	Putative polar amino acid transport system	–4.82
C8J_0951	*livF*	Branched-chain amino acid transport system	–2.01
C8J_0953	*livM*	Branched-chain amino acid transport system	5.44
C8J_0954	*livH*	Branched-chain amino acid transport system	3.80
**Amino acid metabolism**			
C8J_1526	*sdaA*	L-serine ammonia-lyase	–3.03
C8J_0079	*aspA*	Aspartate ammonia-lyase	–4.77
C8J_0666	*glnA*	Glutamine synthetase, type I	–4.99
C8J_0033	*ggt*	Gamma-glutamyltransferase	–9.53
**Fatty acid metabolism**			
C8J_0305	*fabH*	3-oxoacyl-(acyl-carrier-protein) synthase III	2.82
C8J_0417	*fabF*	3-oxoacyl-(acyl-carrier-protein) synthase II	7.42
**C4-dicarboxylate transporter**			
C8J_1136	*dctA*	Putative C4-dicarboxylate transport protein	3.00
**Metabolism of short chain fatty acid**			
C8J_1436	*acs*	Acetyl-coenzyme A synthetase	–4.54
C8J_0069	*lctP*	L-lactate permease	–3.77
**Translation**			
C8J_1498	*rplQ*	50S ribosomal protein L17	2.66
C8J_1605	*rpsC*	30S ribosomal protein S3	–2.20
C8J_0288	*rplY*	50S ribosomal protein L25	–2.09
C8J_0346	*rpsU*	30S ribosomal protein S21	–4.41
**Iron uptake**			
C8J_1312	*feoB*	Ferrous iron transport protein	3.47
C8J_1311	*feoA*	Ferrous iron transport protein	7.63
C8J_1270	*ceuD*	Enterochelin transport system ATP-binding	7.14
C8J_1271	*ceuE*	Enterochelin transport system substrate-binding	2.51
C8J_0419	*cfrB*	Enterobactin transporter	3.95
**Other metal transporter**			
C8J_1438		Tungsten ABC transporter, permease protein	5.64
C8J_0240	*zupT*	Zinc transporter	5.20
**Oxidative stress**			
C8J_0165	*sodB*	Superoxide dismutase (Fe)	–7.35
C8J_0311	*ahpC*	Alkyl hydroperoxide reductase	–3.79
C8J_0335	*ccpA-2*	Cytochrome C551 peroxidase	2.45
C8J_0730	*tpx*	Thiol peroxidase	–5.92
**Efflux pump**			
C8J_0342	*cmeB*	CME efflux system, inner membrane transporter	6.50
C8J_1294		Multidrug resistance efflux transporter	–3.25
C8J_1131	*arsB*	Putative arsenical pump membrane protein	3.83
**Regulator**			
C8J_1205	*racR*	Two-component regulator	–2.87
C8J_1206	*racS*	Sensor histidine kinase	3.39
C8J_1044	*csrA*	Carbon storage regulator-like protein	–3.77
**Adhesins**			
C8J_1383	*cadF*	Outer membrane fibronectin-binding protein	–4.67
C8J_0922	*jlpA*	42-kDa lipoprotein	–3.70
**Flagella**
C8J_0296	*fliG*	Flagellar motor switch protein	2.79
C8J_0312	*flhB*	Flagellar biosynthetic protein	2.31
C8J_0508	*flaG*	Possible flagellar protein	–5.08
C8J_0510	*fliS*	Flagellar protein	–4.85
C8J_0664	*flgG2*	Putative flagellar basal-body rod protein	–4.21
C8J_0665	*flgG*	Flagellar basal-body rod protein	–2.11
C8J_0687	*flaC*	Flagellin	–3.91
C8J_0767	*fliP*	Flagellar biosynthesis protein	2.65
C8J_1368	*flgI*	Flagellar P-ring protein FlgI	–2.84
C8J_1576	*fliQ*	Flagellar biosynthetic protein	3.88
**Glycosylation**			
C8J_1067	*pglJ*	General glycosylation pathway protein	–3.71
**Lipopolysaccharide (lipooligosaccharide) synthesis**			
C8J_1251	*neuB*	*N*-acetylneuraminic acid synthetase	3.40
C8J_0762	*lpxK*	Tetraacyldisaccharide 4′-kinase	4.84
C8J_1095	*gmhA-1*	Phosphoheptose isomerase	4.00
C8J_1096	*waaE*	Putative ADP-heptose synthase	3.50
C8J_0262		UDP-2,3-diacylglucosamine hydrolase	2.74
C8J_0264	*lpxB*	Lipid-A-disaccharide synthase	2.75
C8J_1074	*waaM*	Lipid A biosynthesis lauroyl acyltransferase	2.56
**Peptidoglycan synthesis**			
C8J_0407	*murD*	UDP-*N*-acetylmuramoylalanine-D-glutamate ligase	3.68
C8J_0408	*mraY*	Phospho-*N*-acetylmuramoyl-pentapeptide-transferase	3.94
C8J_0749	*ddlA*	D-alanine-D-alanine ligase	3.47
C8J_0746	*murF*	UDP-*N*-acetylmuramoyl-tripeptide—D-alanyl-D-alanine ligase	–2.73
C8J_0843	*pgp2*	LD-carboxypeptidase	3.79
C8J_1261	*pgp1*	DL-carboxypeptidase	4.14
**Unique genes of 81116**			
C8J_0133		Putative DNA-methyltransferase	2.97
C8J_0398		Protein of unknown function	4.15
C8J_0648		Hypothetical protein	1.63
C8J_1333		Putative CMP-NeuAc synthase	3.32
C8J_1342		Hypothetical protein	4.51
C8J_1423	*cas2*	CRISPR-associated protein Cas2	3.51
C8J_1619		Hypothetical protein	4.75

### Validation of the Expression of Selected Genes by RT-qPCR

**FIGURE 5:**
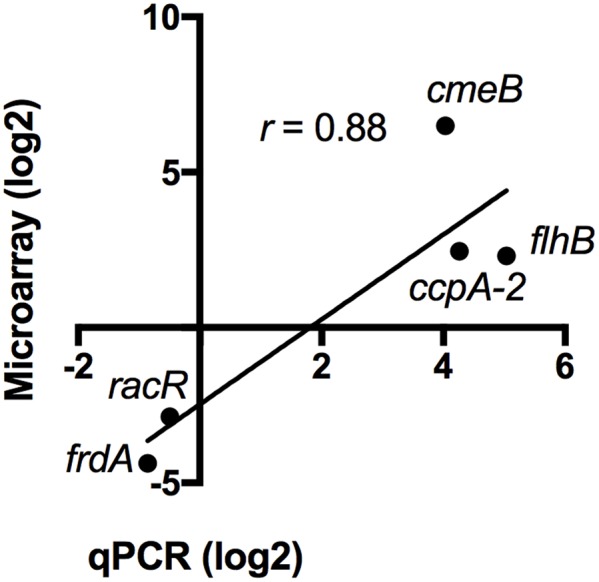
Validation of the transcriptomic data by RT-qPCR. The expression of five differentially regulated genes was validated by RT-qPCR. The values obtained by microarray and by qPCR are shown. The correlation (*r*) between each data set is 0.88.

To validate the transcriptomic data, the expression profile of five genes was confirmed by reverse transcription-quantitative PCR (RT-qPCR) using 16S rRNA as an internal control. The genes tested by RT-qPCR were selected from five different gene ontology groups: energy metabolism (*frdA*), oxidative stress (*ccpA-2*), regulation (*racR*), motility (*flhB*), and multidrug efflux pumps (*cmeB*). Consistent with the microarray data, the RT-qPCR analysis confirmed induction of *ccpA-2, cmeB*, and *flhB*, and repression of *frdA and racR* (**Figure 5**). Overall, the correlation between microarray values and RT-qPCR values is 0.88, which validates the transcriptomic data (Draghici et al., 2006).

### Strain 81116 Expresses Unique Genes in Fraquil

**FIGURE 6:**
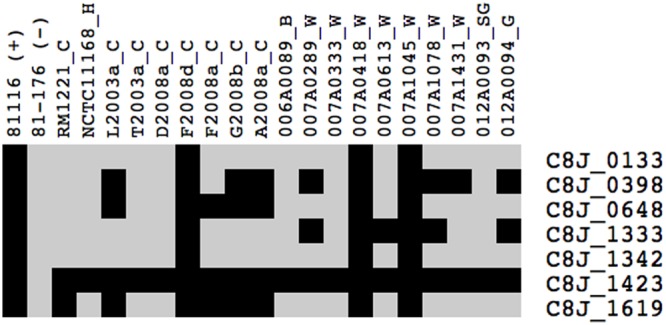
Presence of genes unique to 81116 induced in water in different isolates of *C. jejuni*. The presence of the select genes was tested by PCR using strains 81116 and 81-176 as positive and negative controls, respectively. Black indicates that the gene is present in the strain, whereas light gray indicates that the gene is absent. The letter following the name of each isolate indicates its source of isolation: H, human; C, chicken; B, bovine; SG, snow goose; G, gull; W, water.

Since 81116 survives better in water than strain 81-176, we hypothesized that genes unique to 81116 could contribute to this phenotype. The Pan-genome analysis tool (Miele et al., 2011; Vallenet et al., 2013) on the MicroScope website revealed that the two strains used in this study have 1473 genes in common. Strain 81116 possesses 165 unique genes, while 81-176 encodes 296 unique genes, including those encoded on the pVir and the pTet plasmids (Hofreuter et al., 2006). Twelve genes that are unique to 81116 were strongly expressed in Fraquil compared to rich broth (**Table 3**). Their presence was subsequently tested in 19 *C. jejuni* strains isolated from various water and animal sources. We hypothesized that strains originating from aquatic environments would harbor these unique genes, while those isolated from animals would lack them. Despite being classified as genes unique to 81116, PCR analysis revealed that 5 sets of primers tested amplified a product in 81-176. This is likely due to the amplification of a homologous gene, an annotation error, or low primer specificity. The distribution of the remaining seven genes was tested by PCR using chicken, bovine, human, snow goose, gull and water isolates, as well as the model strains NCTC 11168 and RM 1221 (**Figure 6**). Six genes encode hypothetical proteins or proteins with a putative function, while C8J_1423 codes for a CRISPR-associated protein called Cas2. Four isolates, including 81116, possess the full set of genes tested. None of the genes tested were present solely in water isolates. C8J_1423 was only absent in 81-176, suggesting that the CRISPR system is widely distributed in *C. jejuni* as previously reported (Pearson et al., 2015).

## Discussion

Transmission of campylobacteriosis to humans occurs via consumption of contaminated foods or water. The ability of *C. jejuni* to survive in water determines its ability to be transmitted by water to humans, or from one animal reservoir to another (Bronowski et al., 2014). The ability to survive in water varies greatly between *C. jejuni* strains (Buswell et al., 1998; Thomas et al., 1999; Tatchou-Nyamsi-König et al., 2007; Trigui et al., 2015b), resulting in some sequence types (ST) being isolated more frequently from water than others (Sopwith et al., 2008; Carter et al., 2009; Lévesque et al., 2013). This suggests that some strains are better adapted for utilizing water as a vehicle for transmission. This study aims at identifying phenotypes associated with the ability to survive in freshwater, and determining the transcriptomic response of a strain adapted to survive in water. Since the composition of tap water is variable, the experiments were carried out in the chemically defined freshwater medium Fraquil, which approximates the composition of freshwater of North America (Morel et al., 1975; Trigui et al., 2015b). First, the ability of each strain to survive in Fraquil was assessed using CFU counts and the LIVE/DEAD assay. 81116, a strain isolated during a water-borne outbreak of campylobacteriosis (Palmer et al., 1983), was found to survive in water better than 81-176, an epidemic strain isolated from contaminated milk (**Figure 1**) (Korlath et al., 1985). We hypothesized that the capacity of each strain to survive in different environmental conditions explains their mode of transmission and their respective outbreaks (Bronowski et al., 2014).

In addition, we report that exposure to Fraquil also affects the sensitivity of these strains to oxidative stress and bile salts (**Figures 2, 3**). Oxidative disinfectants, such as chlorine, are routinely used in processing plant chiller water to prevent cross contamination and reduce *C. jejuni* loads on carcasses (Kameyama et al., 2012). Our results suggest that some strains, such as 81116, are better than others at resisting these disinfection procedures during chilling and are more likely to contaminate the final product. A recent study reports that some *flaA* genotypes are selected by the slaughtering process and appear more frequently on the finished product (Kudirkienė et al., 2011).

The resistance of strain 81116 to bile salts was not affected by exposure to Fraquil or rich broth (**Figure 3**). In contrast, 81-176 was markedly more sensitive to this stress (**Figure 3**). The CmeABC efflux pump mediates resistance to bile salt and is essential for colonization of the intestinal tract (Lin et al., 2003, 2005, 2007; Gibreel et al., 2007; Young et al., 2007; Caldwell et al., 2008). While 81116 induced the expression of *cmeB* in response to Fraquil, the remaining components of the efflux system were not differentially expressed in water relative to rich broth, suggesting that this system is expressed at the same level in water and in broth (Supplementary Table S1). Possibly, the expression of this efflux pump is reduced in 81-176, which results in lower resistance to bile salts. *C. jejuni* is unlikely to be exposed to sodium choleate at concentrations used in this study in the natural environment. Nevertheless, the increased sensitivity of 81-176 to bile salts after exposure to water (**Figure 3**) suggests that some strains are better at colonizing the intestinal tract following transmission by water compared to other strains.

To better understand the genetic elements that contribute to the enhanced survival of 81116 in water, its transcriptome in Fraquil was compared to that in Brucella broth. To this end, 81116 was exposed to Fraquil at 4°C since it survives better at this temperature compared to 25°C (**Figure 1**). The exposure time was limited to 4 h because transcriptional changes are known to happen quickly in response to a new condition (Hinton et al., 2004). Moreover, the rate of transcription is reduced dramatically during starvation (Srivatsan and Wang, 2008), as evidenced in *Legionella pneumophila* exposed to water (Li et al., 2015). Since phenotypic differences were observed between Fraquil and rich Brucella broth (**Figures 2, 3**), suspension in rich Brucella broth at 4°C for 4 h was used as the control condition. In addition, this control allows the study of the starvation response of *C. jejuni*, which is likely necessary to survive in the nutrient-poor water environment. Starvation of *C. jejuni* in Ringer solution induces heat-shock resistance and affects the expression of catalases and superoxide dismutases (Klancnik et al., 2009). Our analysis revealed that 336 genes are differentially regulated upon exposure to Fraquil. *C. jejuni* uses amino acids, and to a lesser extent, short chain fatty acids as carbon and energy sources (Stahl et al., 2012). Since Fraquil is devoid of these nutrients, expression of transporters and enzymes involved in their catabolism should be repressed. Indeed, the amino acid transporters *pebA* and *pebC*, as well as the aspartate ammonia-lyase *aspA* and the gamma glutamyltransferase *ggt* required for the utilization of aspartate, glutamate and glutamine were repressed in Fraquil (**Table 3**). The serine ammonia-lyase *sdaA* followed a similar expression pattern (**Table 3**). In contrast, genes involved in the biosynthesis of various amino acids were induced in Fraquil (**Figure 4**). Genes involved in the catabolism of short-chain fatty acid, such as *acs* and *lctP* were also repressed following exposure to Fraquil (**Table 3**); however, *fabH* and *fabF* involved in the biosynthesis of fatty acids were induced.

Costly biosynthetic processes such as glycosylation (*pglJ*) and production of the flagellum (*flaG, fliS, flgG, flgI*) were repressed in Fraquil (**Table 3**). Repression of flagella genes was expected since *C. jejuni* is known to repress them in the post-exponential phase of growth, when nutrients are limiting and waste accumulates (Wright et al., 2009). Adhesins used during infection, including *cadF* and *jlpA* were strongly repressed in Fraquil.

The need to repress costly metabolic systems in water is supported by recent analyses of the survival of *L. pneumophila* in water (Li et al., 2015). *L. pneumophila* is transmitted to humans by inhalation of contaminated aerosols (Mittal et al., 2013). Transcriptomic profiling revealed that *L. pneumophila* represses most systems in Fraquil, including transcription, translation, flagellum synthesis, and virulence factors (Li et al., 2015). In *L. pneumophila*, this is mediated by the stringent response and the sigma factor RpoS (Trigui et al., 2015a). *C. jejuni* does not possess RpoS and only codes three sigma factors (RpoD, FliA, and RpoN) within its genome (Parkhill et al., 2000). RpoD is the housekeeping sigma factor, while FliA and RpoN regulated flagella synthesis and defense against various stresses, respectively (Hwang et al., 2011). The stringent response is initiated by the production of guanosine tetraphosphate (ppGpp), a cellular alarmone signaling starvation (Mittenhuber, 2001). In general, Gram-negative bacteria employ two enzymes to regulate cellular levels of ppGpp; RelA which synthesizes the alarmone and the dual acting SpoT which has low synthase and high hydrolase activities (Mittenhuber, 2001; Gaynor et al., 2005). However, ppGpp levels in *C. jejuni* are regulated solely by SpoT. Deletion of *spoT* affects multiple phenotypes in *C. jejuni*, including interaction with host cells, resistance to rifampicin and oxygen, and survival in the stationary phase (Gaynor et al., 2005). *C. jejuni* survives poorly in stationary phase compared to model bacteria, presumably because it lacks an *rpoS* homolog, the stationary phase sigma factor (Kelly et al., 2001). It is likely that survival of *C. jejuni* in water is mediated mainly by the stringent response. Transcriptomic analysis of the *spoT* mutant at different growth phases in rich broth revealed that 30 genes are regulated by the stringent response in *C. jejuni* (Gaynor et al., 2005). The transcriptome of 81116 in Fraquil showed limited similarities to the *spoT* mutant in broth, which suggests that, in Fraquil, only a few genes are under the control of the stringent response. For example, *clpB* is repressed in Fraquil, but induced in the *spoT* mutant, while and the putative ferredoxin *napH* was induced in Fraquil, but repressed in the *spoT* mutant. Attempts at deleting *spoT* in 81116 in order to confirm the role of the stringent response in water was unsuccessful. Additional studies are required to fully appreciate the role of the stringent response in the survival of *C. jejuni* in water.

Strain 81116 induces 187 genes in Fraquil, which may provide useful functions for its survival. Genes involved in the synthesis of the cell envelope were induced in Fraquil (**Figure 4**), including peptidoglycan synthesis (*ddlA, murD*, and *mraY*) and lipopolysaccharide (lipooligosaccharide) synthesis (lpxB, lpxK, waaE, and waaM). Genes involved in iron transport (*feoA, feoB, ceuDE*, and *cfrB*), as well as other transport systems and porins were also induced. Some of the genes induced in Fraquil code for hypothetical proteins or proteins with a putative function. The potential contribution of the aforementioned genes to the survival of *C. jejuni* in water is discussed in the following paragraph.

The peptidoglycan layer plays a role in maintaining the turgor pressure of the cell, and is required for cell growth and division (Egan et al., 2016). *C. jejuni* cell morphology changes from a rod or spiral shape in the exponential phase to a coccoid form in the stationary phase of growth (Ikeda and Karlyshev, 2012). The amount of peptidoglycan in the coccoid form is about one third of the amount present in the spiral form (Amano and Shibata, 1992). Therefore, *C. jejuni* modifies the amount of peptidoglycan according to growth conditions. The spiral shape is produced by the action of two peptidoglycan modifying enzymes, Pgp1 and Pgp2 (Frirdich et al., 2012, 2014). Both genes are induced in *C. jejuni* upon exposure to Fraquil (**Table 3**). Since *C. jejuni* does not replicate in Fraquil, the induction of peptidoglycan synthesis and modification genes likely serves an ulterior purpose, such as resistance to hypoosmotic stress or differentiation into the VBNC state. The freshwater medium Fraquil used in this study is hypoosmotic relative to Brucella broth. The latter contains 5 g/L of sodium chloride in addition to other osmolytes. *C. jejuni* may induce peptidoglycan synthesis in Fraquil to increase the strength of the peptidoglycan mesh, which in turn, resists the influx of water and maintains cell shape. To our knowledge, the hypoosmotic response of *C. jejuni* has never been studied. Exposure of *C. jejuni* to moderate hyperosmotic stress results in cell elongation and induces the expression of chaperones, oxidative stress response genes and amino acid synthesis genes (Cameron et al., 2012). The present study and others have shown that exposure of *C. jejuni* to water triggers differentiation into the VBNC state (Cools et al., 2003; Baffone et al., 2006; Trigui et al., 2015b). In *E. coli*, the peptidoglycan layer undergoes extensive modification upon entry into VBNC state (Signoretto et al., 2002). Similarly, VBNC *Enterococcus faecalis* cells contain high level of O-acetylated peptidoglycan (Pfeffer et al., 2006). In addition, VBNC *E. faecalis* are more resistant to mechanical stress than actively growing cells, likely due to increased peptidoglycan cross-linking (Signoretto et al., 2000). It is tempting to postulate that induction of peptidoglycan-related genes in Fraquil are necessary to modify the cell wall of *C. jejuni* during differentiation into the VBNC state, protecting cells against stresses, including hypoosmotic stress, encountered in the water environment. The population of *C. jejuni* cells exposed to Fraquil enters the VBNC state progressively (**Figure 1**); however, the prerequisite cellular modifications likely occur relatively quickly after exposure to Fraquil, when cells have sufficient energy and supplies to do so.

*C. jejuni* lipooligosaccharide (LOS) consists of a lipid A moiety, an inner core composed of a conserved trisaccharide and a strain-variable outer core consisting of various sugars (Karlyshev et al., 2005). LOS is similar in structure and function to lipopolysaccharide, but lacks the O-antigen. Modification of LPS is important for many pathogens to evade the host immune defenses (Whitfield and Trent, 2014). Similarly, mutations affecting the length of the outer core of LOS in *C. jejuni* reduce resistance to complement-mediated killing and colonization of mice (Naito et al., 2010). In addition, abnormal LOS results in increased susceptibility to polymyxin B and sodium dodecyl sulfate, but increase biofilm formation (Naito et al., 2010). The effect of temperature on LOS length is strain-dependent, but growth at 42°C favors the production of a shorter LOS (Semchenko et al., 2010). Induction of LOS synthesis genes in Fraquil suggests that modification of the LOS sheath is important for survival in water and/or for differentiation into the VBNC state.

Genes involved in the acquisition of iron were induced in Fraquil, including the ferrous iron transporters *feoAB* and the siderophore transporters encoded by *ceuDE* and *cfrB*. Presumably, the iron-poor environment of Fraquil leads to induction of genes encoding functions related to iron homeostasis. Oxidative stress resistance genes are repressed by iron in *C. jejuni* during growth in minimum essential medium (Palyada et al., 2004; Butcher and Stintzi, 2013); however, they are induced during the metabolic switch from acetate production to acetate uptake, and also between the exponential phase and the stationary phase (Wright et al., 2009). Therefore, it was expected that genes involved in the oxidative stress response would also be induced in Fraquil, since it mimicks the low nutrient condition that is found in stationary phase. Unexpectedly, we found that strain 81116 represses three oxidative stress defense genes, *sodB, ahpC*, and *tpx* in Fraquil. The absence of nutrient and toxic metabolic waste in Fraquil compared to the stationary phase in broth probably leads to the repression of oxidative stress response genes, which could explain the discrepancies with previous studies. Since iron mediates the formation of reactive oxygen species (ROS) inside cells (Imlay, 2003), it is expected that oxidative stress would be reduced in an iron-limiting condition. This speculation is supported by the findings from Folsom et al. (2014) where several enzymes associated with oxidative stress and ROS in *E. coli* were down-regulated under iron limitation, including superoxide dismutases (Sod). Nevertheless, despite the down-regulation of these oxidative stress defense genes in Fraquil, strain 81116 is more tolerant to H_2_O_2_ stress than in rich broth (**Figure 2B**). Interestingly, strain 81116 induces the expression of *ccpA-2*, which encodes for a cytochrome peroxidase enzyme (Kim et al., 2015). It has been shown that loss of *ccpA-2* in *C. jejuni* NCTC 11168 resulted in increased sensitivity to H_2_O_2_ compared to the wild-type (Flint et al., 2014), suggesting that the contribution of *ccpA-2* to oxidative stress response is strain-dependent. Strain 81-176 is more sensitive to oxidative stress in Fraquil than 81116, suggesting differential expression of genes involved in resistance to oxidative stress.

A fraction (32 genes, 9.5%) of the differentially expressed genes codes for hypothetical proteins without known or putative functions. Of these, 10 were induced in Fraquil and could encode functions necessary for 81116 to survive in water. In addition, seven genes induced in Fraquil are absent in 81-176 genome, which suggests that they may contribute to 81116’s ability to better survive in water. Therefore, their presence in water-isolated strains, as well as strains isolated from other sources was tested. The presence of the genes was not correlated with the source of the strain (**Figure 6**) suggesting that the unique genes tested did not contribute significantly to its survival. Alternatively, enhanced survival of 81116 in Fraquil is likely due to the induction of multiple regulatory systems that promote adaptation to water. This is likely due to subtle difference in the regulation of gene expression. Indeed, Dugar et al. (2013) reported that strain-specific transcriptome structure could modulate phenotypic variation among *C. jejuni* strains. This could be due to acquisition of specific regulators, effectors or organization of the genome.

## Conclusion

The transcriptomic profiling of 81116 in water suggest that its ability to survive in water for extended periods of time is due to multiple adaptations that shut down nutrient uptake systems, and costly metabolic pathways, including synthesis of the flagellum. Moreover, the induction of stress response pathways, genes involved in detoxification and cell wall synthesis in response to water enhances the resistance of 81116 to multiple stresses in the aquatic environment. Unique genes do not contribute to its enhanced fitness. Strains with a similar genetic background as 81116 are likely better at transmission via water and more resistant to current disinfection processes.

## Author Contributions

HT, AT, PF, AL, and SF designed the experiments. HT, KL, and AT performed the experiments. AT, SL, PF, and AL provided strains of *C. jejuni*. HT, AT, NM, and SF analyzed the data. HT, NM, and SF wrote the manuscript. All authors edited the manuscript and approved the final version.

## Conflict of Interest Statement

The authors declare that the research was conducted in the absence of any commercial or financial relationships that could be construed as a potential conflict of interest.
